# Kin relationality and ecological belonging: a cultural psychology of Indigenous transcendence

**DOI:** 10.3389/fpsyg.2023.994508

**Published:** 2023-10-20

**Authors:** Yuria Celidwen, Dacher Keltner

**Affiliations:** ^1^Department of Psychology and Othering and Belonging Institute, University of California at Berkeley, Berkeley, CA, United States; ^2^Department of Psychology, University of California at Berkeley, Berkeley, CA, United States

**Keywords:** Indigenous sciences, ethics of belonging, kin relationality, ecological belonging, cultural psychology, self-transcendence, compassion, awe

## Abstract

In this article, we consider prosociality through the lens of an Indigenous “ethics of belonging” and its two constitutive concepts: kin relationality and ecological belonging. Kin relationality predicates that all living beings and phenomena share a familial identity of interdependence, mutuality, and organization. Within the value system of ecological belonging, an individual’s identity is constituted in relation to the natural environment, centered on the sentiments of responsibility and reverence for Nature. We detail how Indigenous perspectives upon prosociality differ from Western scientific accounts in terms of the motives, scope, and rewards of altruistic action. Grounded in this understanding, we then profile three self-transcendent states, compassion, gratitude, and awe, and their similarities across Indigenous and Western approaches, and how kin relationality and ecological belonging give rise to cultural variations. We consider convergent insights across Indigenous and Western science concerning the role of ritual and narrative and the cultural cultivation of kin relationality and ecological belonging. We conclude by highlighting how these two core concepts might guide future inquiry in cultural psychology.

## Introduction

The current international working designation of Indigenous Peoples characterizes these identities as having endured historical colonization and invasion of their Lands (Martinez Cobo, 1981) and extraction and exploitation of their natural resources – air, water bodies (from ice to oceans), mountains and forests, and all that lives in them (United Nations, 2013). Despite these circumstances and the growing threats of the climate emergency, the world’s five thousand Indigenous cultures have formed a resilient political coalition against human and environmental rights violations. Although Indigenous individual and collective rights have only recently been acknowledged in 2007 (United Nations, 2007), Indigenous cross-cultural accord has allowed the emergence of an influential relational identity in a global community beyond just a political one (Wildcat and Voth, 2023). These identities manifest in Indigenous psychologies distinct to each Nation and that can be understood within a framework of Indigenous relationality (Tynan, 2021; Wildcat and Voth, 2023).

Our inquiry seeks to understand the forms of Indigenous relationality as models of human prosociality and self-transcendent emotions. We do so from the perspective of Indigenous sciences and the broader thesis that Celidwen has begun to chart in the “ethics of belonging” (Celidwen, 2020a,b,c). The ethics of belonging differ from the Western view of *homo economicus*—or economic human—which assumes that the natural state of humans is competitive, transactional, narrowly selfish, and oriented toward maximizing individual pleasure (Rittenberg and Tregarthen, 2013). Within the framework of the ethics of belonging, two foundational concepts are germane to a new look at human prosociality: kin relationality and ecological belonging.

One might think of these two Indigenous constructs as constituting a prosocial dimension of human nature organized by a set of assumptions concerning human emotion, cognition, motivation, and action oriented toward collectivism and self-transcendence. We might call this dimension of humanity *ch’ul jkanan* “steward of the sacred” or *Kanan k’inalat* “protector of Mother Earth” in Maya Tseltal, or *homo reverens* or “reverential human” in Latin.^1^

We ground this work committed to epistemological equity. By this, we mean considering Indigenous and Western ways of knowing as based on scientific inquiry, with systematic methods of gathering evidence and assessing beliefs about social and physical reality. Both forms of knowing constitute science within culturally specific practices of rigorous observation, analysis, and evaluation that encourage and advance learning, discovery, and comprehension of the world. These ways of knowing, scholars in different disciplines have observed, emerge from context- and culturally-specific interests and concerns (Medin et al., 2013; Larsen et al., 2017; Bang et al., 2018; Gone, 2021; Celidwen, 2023).

Western science epistemology is instantiated in formal theories, hypotheses, and testing oriented toward falsification and universalization. Critiques of this learning method center upon the tendency for Western science toward essentialism, and its problematic assumptions that what is learned is value- and culture-free (Bang et al., 2018). In Indigenous sciences, on the other hand, systematic understanding is often arrived at through contextual narratives of the self and the collective, and symbolic, mythic relationships and imagination. These practices draw upon multi-generational experiences of “deep spatial” traditional ecological knowledge of landscapes and seascapes (Wildcat, 2013). They center on what Tuck and MacKenzie call relational validity: prioritizing place in a contextual interconnectedness of human life to Lands and all other living beings (Tuck and McKenzie, 2015). Accordingly, Indigenous sciences center these relationships in contexts of cosmologies and sovereignty. Thus, colonialism and its continuous devastating effects on shaping knowledge, cultural identities, and relationships are reckoned as annihilating disturbances of identity.

Thus, this essay bridges multiple forms of empirical evidence, from the cultural and ethnographic to the neurophysiological. In considering these different disciplines, here we center on (1) the deep cultural study of the role of kin relationality and ecological belonging within the world’s Indigenous sciences and their breadth of critical place inquiry and (2) recent advances in the study of self-transcendent states—compassion, gratitude, and awe—through cultural evolution. This inquiry leads us to present a first cultural understanding of kin relationality and ecological belonging.

In the ensuing sections, we will discuss in greater detail what kin relationality and ecological belonging entail. To anticipate this discussion, kin relationality refers to the Indigenous ontologies based on relationships, in which living beings and phenomena are recognized as part of a family lineage. In such conceptions, each being and phenomenon shares a familial bond of group identity of interdependence, mutuality, and organization. In kin relationality all of existence is deemed a Relative.

Ecological Belonging conceptualizes the self as belonging to a kin relational, collective Earth system network—its ecosystems and life cycles. It manifests as a recognition of belonging to a planetary identity in an orientation toward benefiting life forms and the natural world through affect (compassion toward living forms), cognition (recognition and acknowledgment of the interdependence of living forms), volition (intention and responsibility for the Earth as a network of beings and phenomena), and motivation (stewardship for thriving of that community of beings and phenomena). In ecological belonging all of existence belongs to environmental systems and networks.

Within these two concepts, the self and the collective exist within patterns of mutual influence in kin relational networks. Such interactions resemble classic treatments of interdependence (Markus and Kitayama, 1991). Thought, feeling, and action are constituted in relationships within groups (Trafimow et al., 1991). As our essay progresses, we shall highlight distinct dimensions of Indigenous interdependence: that the self is not viewed as being in a relationship with other people or things but as being the relationships themselves (Wilson, 2008); that such identity-based meaning-making involves dynamic, ongoing relations with Lands and ecosystems and their specific phenomena (TallBear, 2013; Booth, 2015). Indigenous interdependence is deeply local, arising out of contextualized sense-making, in particular in relation to the land; at the same time, Native perspectives worldwide share remarkably similar views in conceiving the Earth as a Mother and all creation as related, and a shared sense of kin and caring for the environment.

In considering these two concepts, we offer a dialogue with Western conceptions of prosociality, focusing on how kin relationality and ecological belonging transform the self-transcendent states of compassion, gratitude, and awe and the scope of prosocial behaviors to which these states can lead. We then consider the convergence in Indigenous sciences and recent theorizing about cultural evolution concerning how stories, rituals, and ceremonies promote the cultivation of the ethics of belonging through transcendent states. We then close with a consideration of how the ethics of belonging might shape a next chapter in cultural psychology.

## Indigenous prosociality within the ethics of belonging: kin relationality and ecological belonging

The many forms of prosociality—sacrifice, sharing, and compassion—were once considered “problems” within Western social sciences (Schwartz, 1987). That model of economic human interactions—a *homo economicus* account of prosociality—has been challenged empirically by a multidisciplinary field focusing on promoting the welfare of others (Katz, 1999; Keltner et al., 2014; Allen, 2018; Dahl and Davidson, 2019; Pressman et al., 2019; Dunne and Manheim, 2022; Keltner, 2023). These shortcomings of a narrow self-interest account of human behavior serve as a point of departure for Indigenous perspectives on transcendence and prosociality.

For example, studies have revealed that young children routinely help strangers in need, beginning in the first 18 months of life (Svetlova et al., 2010; Warneken and Tomasello, 2015). In over 25 cultures worldwide, people typically share 40 to 50% of a resource with a stranger when asked. People share more when making such decisions quickly (Recalde et al., 2017). And sharing resources can be an effective way to deal with environmental risks (Suleiman et al., 2015) by strengthening social bonds (Bird-Naytowhow et al., 2017). These prosocial tendencies are supported by physiological networks in the brain and below the brain stem (Keltner et al., 2014), as we detail below (see also Park et al., 2017).

Most generally, Indigenous societies tend to orient toward community models of ethical living and shared allocation of resources (Corr, 2002; Acosta and Martínez, 2009; Celidwen, 2020b), aligning with the framework of the “ethics of belonging” and its two central concepts of kin relationality and ecological belonging. In practice, these core concepts denote an ethos that manifests in patterns of thought and emotional experience that are oriented toward mutual aid (Gonzales and Husain, 2016), participatory action (Cajete, 2021), and ethical pursuits favorable to the health of humans (Celidwen et al., 2023), lands and territories (Fa et al., 2020), all other communities of living beings (Salmón, 2000), and consequential for a healthy planet (Redvers et al., 2022). The condition of a healthy environment is now considered a human right (Andorra et al., 2022).

*Kin relationality* commonly appears in Indigenous belief systems worldwide as a way of conceptualizing and expressing relationships. This form of relationality registers in ontologies (ways of being) that connect living beings with larger collectives or systems, such as Lands, local ecosystems, and cosmic systems of meaning. It is experienced, for example, in how individuals locate their individual identities within origin stories or cosmogonies about the coming of being of the universe, and the culture within that universe (Brennan and Ungunmerr-Baumann, 1989; Cochoy Alva, 2006; Johansson, 2015; Cajete, 2017; Eickelkamp, 2017; Tynan, 2021). Within this model of relationality, living beings and natural phenomena share a familial identity of interdependence and mutuality that conceives relationships as continuously influenced, adapted, developed, and organized from interactions (Gratani et al., 2016; Nalau et al., 2018; Celidwen, 2020b). Such relational systems are believed to replicate in life systems (Cajete and Little Bear, 2000; Ramos, 2012; Redvers et al., 2020; Goodchild, 2021; Celidwen, 2022b).

*Ecological belonging* centers upon the conceptualization of the Indigenous self-construal—or perception of one’s identity—as belonging to a responsive collective ecosystem. Thus, ecological belonging is grounded in a collective identity based on what has been called a planetary group-consciousness (Cajete and Little Bear, 2000; Nelson and Shilling, 2018; Redvers et al., 2022; Celidwen, 2022b; Celidwen et al., 2023). Within the value system of ecological belonging an individual’s identity is constituted in relation to the natural environment (Brennan and Ungunmerr-Baumann, 1989; Cajete, 2009; Mann, 2016; Celidwen, 2020b,c)—including spaces not customarily defined as untamed, like urban environments. Ecological belonging thus differs from concepts such as “deep ecology” or the “ecological self,” which presuppose subject-object relations between humans and the natural world (Mathews, 1994; Teubner et al., 1994; Drengson and Inoue, 1995; Smith and Williams, 1999).

Kin relationality and ecological belonging are fundamental to the Indigenous Nations worldwide—making up 6% of the global population – for cultivating inter and intra-species community bonds and a clear sense of environmental personhood (Youatt, 2017; O’Donnell et al., 2020; Poelina et al., 2020). In the exemplary cases of Bolivia, Ecuador, New Zealand, and Uganda—countries with large Indigenous populations—these philosophies have been included in their federal legislation (República del Ecuador, 2008; Plurinational State of Bolivia, 2011; Te Urewera Act 2014 No 51, 2014; National Environment Act, 2019). The Indigenous global movements for the defense of the rights of governance and stewardship in ancestral Lands have made the most of the plurality of multi-ethnic distinctions to create successful participation platforms through horizontal and democratic structures (Acosta and Martínez, 2009; Inter-American Commission on Human Rights, 2009; Ramos, 2012; Postero, 2017; Oliva Martínez, 2022). A common agreement of this Indigenous political identity unites shared values like kin relationality and ecological belonging (McDermott, 2000; Cochoy Alva, 2006; Neeganagwedgin, 2013; Turner, 2014).

In their shaping of social, political, and economic life, kin relationality and ecological belonging constitute an overarching ethical model of reverence that reveals environmental systems and relationships as having sentience, distinctness, and agency (as also suggested by Poelina et al., 2020; RiverOfLife et al., 2020). These concepts have inspired the influential movement toward planetary health (Ford, 2012; Nalau et al., 2018; Redvers, 2018; Ratima et al., 2019; Redvers et al., 2022). This form of environmental welfare is the understanding of health and flourishing of all living forms, including environmental occurrences, extending as well to cultural phenomena such as cosmogonies (Johansson, 1997; Cochoy Alva, 2006; Mazariegos, 2011), rituals and rites of passage—including end of life transitions (Austin, 1960; Lhuillier, 1963; Johansson, 2003)—that connect humans with their environment and with past and emerging ancestry.

Kin relationality and ecological belonging are endorsed and supported through spiritual pursuits embedded in cultural expressions in oral, embodied, and collective narratives (for example, in languages, storytelling, habits, rituals and rites of passage, songs, or ceremonial dances, law and governance, and lifeways). Most often, these narratives intend to nourish awareness of “Spirit,” defined here as the animating principle of life weaving all relationships (Celidwen, 2020c). In Table 1, we highlight this Indigenous view of prosociality, and summarize a Western scientific view we turn to shortly, to reveal sharp contrasts in Indigenous and Western culturally situated perspectives.

**Table 1:** A summary of Indigenous and Western scientific perspectives upon prosociality.

	Indigenous kin relationality and ecological belonging	Western *homo economicus*
Core motive	Advance health and flourishing of the planetary system	Advance personal pleasure
Scope of prosociality	All forms of life (social and environmental systems)	Biological kin and individuals within transactional relationships and groups
Nature of reward	Gratification at collective flourishing	Pleasure at personal gain achieved through the reduction of distress and enhanced social reputation

With respect to cognitive tendencies of kin relationality and ecological belonging, individuals are not separate and independent, but are perceived as in constant relationship with all beings and phenomena, as noted above. The relationships that ceaselessly shape identity include humans and beings other than human, living and deceased, from generations of Ancestors to Lands, ecosystems, natural phenomena, and the mere elements of which matter is made of. Ancestors have a spiritual quality that transcends time and transmutes form. Hence, they are past, present, or yet to come, and exist in matter and subtle energies, the latter understood as spiritual presences or animic entities. Lineage is regarded to have a profound influence on sense-making, inclusive of outlining aspects of policy on ancestral domains, habits and customs, governance and law, and community-based resource management (McDermott, 2000; Youatt, 2017; O’Donnell et al., 2020; RiverOfLife et al., 2020; Celidwen, 2020b).

From these viewpoints, Lands, ecosystems, and the planet are co-creators of systemic Indigenous worldviews (Wildcat, 2022). As such, they would be as well co-creators of cognition and emotion. Moreover, they emphasize not only interpersonal relationships, but also intersomatic processes, and spiritual counterpart relations (Groark, 2013). Therefore, humans become accountable and act in reciprocal ways within the familial bonds they share with the entire environment, sharing in identity in terms of interdependence, mutuality, organization, and collaboration. Recent measures of relatedness to Nature are beginning to capture these notions (Nisbet and Zelenski, 2013; Dean et al., 2018; Grabowska-Chenczke et al., 2022). This form of broader kinship is central to efforts to decolonize psychological interventions, counseling, and clinical practice, marking a shift to the therapeutic reclamation of Indigenous psychological expertise (Almaguer González et al., 2014; Hartmann et al., 2019; Gone, 2021).

Self-transcendent states core to kin relationality and ecological belonging are reverence, compassion, gratitude, awe, and love. All are evoked by and oriented toward all living forms and natural phenomena in Indigenous ethical notions of good living and reciprocity. These self-transcendent states are woven into patterns of Indigenous social organization and cosmovisions—or deep theories about the structure and origin of life. Examples of this are the Indigenous Maya *Ut’z Kaslemaj* (Elías, 2020), the Andean Quechua pursuit *Sumak Kawsay* (Lema et al., 2011) and the Aymara *Sumaq Qamaña* (Ogawa, 2017), the Guaraní *Teko Porã* (Melià, 2015), the Māori *Hauora* (Te One and Clifford, 2021), and others.

Around the globe, for Indigenous Peoples, who have endured extreme environments—now more severely endangered by the climate emergency—ensuring the availability of resources has depended on maintaining relationships and stewarding the landscapes, inclusive of all other-than-human relationships in them. The bases for jurisprudence systems such as Natural or First Law tend to such relationships with the Land (Yotti Kingsley et al., 2009; O’Donnell et al., 2020; Redvers et al., 2020). Thus, interdependent relations are embedded in the beliefs, practices, and values of kin-relationality and ecological belonging, and the sense of responsibility for all living beings they entail. Understanding natural relationships is a matter of survival for Indigenous peoples (Smith and Williams, 1999; Arsenault et al., 2019; Alessa, 2020; Fa et al., 2020).

In Table 1, we juxtapose this Indigenous conception of prosociality with that which has emerged in Western science. Building upon earlier considerations of reciprocal altruism and kin selection, a recent review highlights five core principles of prosociality (Nowak, 2006). These principles include how humans show tendencies of prosociality toward kin (biological kin selection), towards non kin within social networks through simple processes of reciprocity (direct reciprocity) and towards others who are likely to benefit the self in the future (known as indirect reciprocity and network reciprocity), and toward members of one’s own groups or tribes (group selection).

We note that in Western scientific approaches, little if any mention has been given to human prosocial tendencies toward other-than-human species, which is a centerpiece of kin relationality and ecological belonging, and the orientation of self-transcendent states toward all living forms. As we suggest in Table 1, Indigenous and Western perspectives depart in critical ways:

1. They diverge in terms of claims about the *core motives* animating prosocial behavior (for example, the advance of the planetary system in Indigenous approaches versus personal pleasure or rewards).
2. They differ in their assumptions about the *scope* of prosociality (to all living forms, including nonhuman life forms, versus a focus on close biological kin and transactional relationships).
3. They depart considerably in thinking about the *rewards* of prosocial behavior (gratification at the flourishing of the collective versus the personal pleasure of prosocial action).

Within both Indigenous and Western perspectives on prosociality, emotions like compassion, gratitude and awe are widely assumed to be proximal determinants of the different forms of prosociality (Batson et al., 2005; Goetz et al., 2010; Keltner et al., 2014; Schroeder and Graziano, 2015; Celidwen, 2020a). In keeping with central theorizing in cultural psychology (e.g., Markus and Kitayama, 1991), the framework we offer here anticipates striking cultural variations in these self-transcendent emotions, to which we now turn.

## The grounding of kin relationality and ecological belonging in self-transcendent emotions: Indigenous and Western scientific perspectives

The cultivation of kin relationality and ecological belonging via self-transcendent emotions is central to the foundations of Indigenous sciences. These scientific methods manifest as cultural ontologies and epistemologies, shaping the practices of origin stories, rituals, habits, law and governance, ceremonies, and lifeways, as noted. Thus, studying self-transcendent emotion is a long overdue opportunity to diversify the epistemologies and methods of psychological science. As our work advocates for epistemological equity to decolonize the ways of understanding about cultural psychologies, we draw on states of being and ways of learning from the world’s Indigenous cultures and how they relate to Western systems. In recent years, innovations in methodologies that center Indigenous sources over broad geographical range and traditions and across disciplines have empowered and reclaimed Indigenous expertise and scientific contributions (Denzin et al., 2008; Smith, 2012; Andersen and O’Brien, 2016; Lokensgard, 2018; Chilisa, 2019; Gould et al., 2019). The methods of Indigenous and Western sciences diverge in striking ways and can complement each other in promising new areas of inquiry.

Indigenous sciences explain the world through narratives of observations, evaluations, and documentation of phenomena in a non-linear, qualitative way (Castellano, 2000; Wilson, 2008; Kovach, 2009; Iseke and Brennus, 2011; Iseke, 2013). Narratives are individual and cultural semantic processes, giving meaning to the personal and collective responsibilities toward planetary flourishing. We delineate these narratives into four categories: oral (storytelling), embodied (ritual), collective (ceremonies), and juridical (law and governance). The foci are the narrative’s relational, intersubjective, and communal aspects, showing their contextual and place-based cultural diversity. These methods intend to understand the reciprocal characteristics of phenomena and their sentience, distinctness, and agency.

Western science expresses ideas about causality through theories, hypotheses, and empirical evidence that evaluate and verify through statistical analysis. However, its scope is linear, or cumulative, method-based on replicable quantitative measurement and statistically-driven inferential processes. While this method reveals central, often universal, tendencies, its inattention to context, place, and cultural diversity is of concern. For example, its bases on WEIRD—an acronym for Western, Educated, Industrialized, Rich, and Democratic (Henrich, 2020)—samples renders Western science, from one perspective, colonial in origin. Through this utilitarian lens, empirical studies can have economic, transactional motives, and knowledge is often viewed in terms of ownership (e.g., intellectual property).

Despite their scope and methodological differences, this essay emphasizes the convergence of Western and Indigenous sciences in their interest in self-transcendent states. Namely, these are states of emotion that orient thought and action that lead the individual to integrate into social collectives (e.g., Stellar et al., 2017). Within this vein of thinking, humans thrive physically and mentally in interdependent relationships, like attachments and group membership. A return to Indigenous cultural approaches to therapy have restored a sense of belonging to social collectives and community relationships (Gone, 2013; Brave Heart et al., 2016; Hartmann et al., 2019; Blacklock et al., 2020). Critical to these relationships is the task of shifting away from satisfying narrow self-interest, to an orientation to the needs and interests of others. Emotions like compassion, gratitude, and awe focus individuals on the needs and interests of others while quieting the voice of narrow self-interest (Sober and Wilson, 1998; Hrdy, 1999; Tomasello, 2021; Keltner et al., 2022). These self-transcendent emotions shift patterns of thought and initiate forms of action that benefit others, from soothing to sharing, all supported by shifts toward more tend-and-befriend patterns of physiology.

Compassion is the feeling of concern for another’s suffering accompanied by the motivation to help (Lazarus, 1991), and can be directed to various targets, ranging from others who suffer emotionally to those in immediate danger. Compassion is part of a family of states such as pity, sympathy, and empathic concern, which vary according to secondary appraisals, such as who is the appropriate recipient of generosity (Goetz et al., 2010). Compassion differs from empathy, the sharing of another’s feelings (affective) and understanding of their perspective (Cox et al., 2012); empathy can refer to many shared emotional states such as joy, embarrassment, or sadness (Royzman and Rozin, 2006).

Within Western approaches, compassion motivates the caretaking of offspring and promotes cooperation with non-kin (Goetz et al., 2010). Guided by this framework, empirical studies find that feelings of compassion lead to greater sharing, sacrifice, and philanthropy (for review, see Goetz et al., 2010). Individuals vary in their compassion-driven prosocial tendencies as the result of early attachment experiences (Mikulincer and Shaver, 2005), and cultural factors such as perceived similarity or group relatedness (Batson et al., 2005; Oveis et al., 2010).

Although compassion likely originated to facilitate caring for infants, it extended beyond offspring to promote cooperation between non-kin (Trivers, 1971; Darwin, 2004). This assertion is very much in keeping with the Indigenous concept of kin relationality, as considered earlier, and the notion that prosocial tendencies can be extended in scope. On this, empirical studies find that compassion reduces perceived psychological distance (Oveis et al., 2010) and motivates greater generosity (Saslow et al., 2013), helping (Eisenberg et al., 1989), and more costly forms of aid, such as taking painful shocks in place of another person (Batson et al., 1981).

In keeping with claims that compassion serves evolutionarily significant social functions, compassion-like behavior has been observed in humans’ closest primate relatives (e.g., de Waal and Aureli, 1996) and in the small-scale societies that likely resembled the small groups *homo sapiens* evolved in for several hundred thousand years (Eibl-Eibesfeldt, 1989). The experience of compassion is also communicated across different cultures through patterns of touch (Hertenstein et al., 2006) and vocalization (Cordaro et al., 2016).

Recent neurophysiological studies on compassion, gratitude, and awe suggest that these three self-transcendent states do appear to activate branches of the parasympathetic autonomic nervous system long believed to promote social connection and openness to others (Taylor, 2006; Porges, 2009; Stellar et al., 2015; Gordon et al., 2016). With respect to the central nervous system, one early study in this literature found that compassion, but not pride, is associated with increased activation in the periaqueductal grey, a midbrain structure theorized to support caregiving behavior (Simon-Thomas et al., 2012). More recently, Singer and colleagues have documented distinct neurophysiological patterns for empathy and compassion (for review, see Singer and Klimecki, 2014). Germane to our interest here, compassion is associated with activation of reward-related regions of the brain, including the Ventral Tegmental Area, the Medial Orbital Frontal Cortex, and the striatum (Bernhardt and Singer, 2012; Ashar et al., 2017).

If compassion is oriented toward tending to the needs of those who are vulnerable, gratitude, a second transcendent emotion, arises out of appraisals that one has benefited from the costly, intentional, voluntary action of another person. Gratitude is part of a family of states that includes related states such as appreciation and reverence (McCullough et al., 2000). It is thought, within Western accounts, to solve problems related to resource sharing by motivating patterns of reciprocity (Trivers, 1971; McCullough et al., 2008).

Rudimentary forms of gratitude have been observed in chimpanzees in the context of food sharing, which is targeted toward non-kin who have offered a pleasurable social reward—grooming—at an earlier time (Bonnie and de Waal, 2004; Darwin, 2004). Studies of humans find that gratitude increases the likelihood that the recipient will behave prosocially toward the benefactor in the future (McCullough et al., 2008). These effects can endure for months (Algoe et al., 2008). Compared to other positive emotions, eliciting gratitude by having a confederate help led participants to behave more prosocially toward the confederate in subsequent interactions (Bartlett and DeSteno, 2006). Strong expressions of gratitude elicit greater economic giving (Rind and Bordia, 1995) and helping (Grant and Gino, 2010) in others. These effects extend to many relationships, from strangers to romantic partners to friends (Gordon et al., 2012).

Gratitude also motivates “upstream altruism”—future prosocial behavior toward novel others, thus promoting prosocial tendencies in social networks over time (Nowak and Roch, 2007). For instance, participants induced to feel gratitude were more likely to help a stranger than participants who felt other positive states (Bartlett and DeSteno, 2006). In addition, keeping a gratitude journal each week increased people’s feelings of connection to others, in keeping with conceptions of kin relationality, and reported prosociality compared to control activities (Emmons and McCullough, 2003).

Gratitude appears in various cultures (McCullough et al., 2001). As such, experiences of gratitude are reliably communicated to others via specific patterns of tactile contact (Hertenstein et al., 2006) and verbal responses (e.g., “thank you” (Grant and Gino, 2010). These findings dovetail with earlier theoretical accounts of gratitude, suggesting that it creates more cohesive groups through the strengthening properties of reciprocal or mutual prosociality (Smith, 1759).

When comparing these Western scientific findings to observations of Indigenous cultures, we note a difference: not only is gratitude and reverence felt most deeply toward Nature in Indigenous cultures (little studied in Western perspectives on gratitude), but from an Indigenous perspective the rewards or future return of benefits from Nature are invariably uncertain. In other words, environmental gratitude consistently responds to gifts already received rather than expected, suggesting that within Indigenous perspectives gratitude is more about what has been given than what is anticipated from others in the future. This notion warrants empirical attention.

The neuroscientific study of gratitude is more limited. State-focused studies have had participants practice gratitude or imagine themselves being the recipient of gifts gratitude; individual difference oriented studies have focused on trait-like tendencies to feel gratitude (Brown and Wong, 2017; Henning et al., 2017). This research finds that gratitude tends to be associated with activation in the medial prefrontal cortex, a brain region involved in self-representational processes, which is sensible given that assessments of reciprocity between self and others are central to experiences of gratitude (Fox et al., 2015; Brown and Wong, 2017).

Awe, a third self-transcendent emotion, is experienced when people encounter vast mysteries they cannot immediately understand (Keltner and Haidt, 2003). People feel awe in response to the moral beauty of others, collective action, nature, music and art, ideas about the Divine, and reflections upon the life and death cycle (Shiota et al., 2007; Keltner, 2023). Awe is often likened to wonder—the state of curiosity and exploration that follows feelings of awe—and related states such as admiration, inspiration, and elevation. Although roughly one-quarter of awe experiences elicit fear (Gordon et al., 2017), the majority of awe experiences are positively valenced.

Approximately half of all awe experiences arise in response to other-focused appraisals (the actions of others related to kindness, courage, virtuosity, magnanimity, and physical or psychological stature); the next largest category of elicitors is nature (Shiota et al., 2007). Recent research from other cultures—such as China—suggests the proportion of awe experiences elicited by others is even higher (closer to 75%) in the Global South (Stellar et al., 2017). In light of conceptions of kin relationality, we would anticipate this cultural difference to emerge in studies of Indigenous awe, that it is more oriented toward other humans—family members, family histories, members of collectives, and representations of others in stories or as spiritual beings.

Western scientific approaches highlight how awe promotes stronger groups and is predicated on the assumption that individuals attain goals (e.g., caring for vulnerable offspring, hunting large mammals) and fend off threats (e.g., warfare) more successfully in cohesive groups than alone (Sober and Wilson, 1998; Dunbar and Shultz, 2007; Nowak et al., 2010). Experiences of awe promote more cohesive groups through the “small self” effect (Bai et al., 2017): momentary experiences of awe bring about less of an awareness of the self, narrowly defined, and an increased awareness of the virtues of others (Shiota et al., 2007; Stellar et al., 2018); and a more salient sense of both kin relationality—recognizing familiarity with all of existence—and of ecological belonging, namely that the individual is part of a collective, nature-based identity.

Awe also leads to greater loyalty, willingness to sacrifice, and positive views of the group to which the person belongs (Stellar et al., 2017). Like other self-transcendent emotions, feeling awe predicts prosocial behavior, including generosity in economic games and donation of time to help others (Rudd et al., 2012; Piff et al., 2015). In keeping with arguments we offered earlier, we would expect that awe would promote generosity in Indigenous cultures, as in the West, but the scope of that generosity (i.e., who it is oriented to) would be shaped by the concepts of kin-relationality and ecological belonging (see Table 2).

**Table 2:** Kin relationality and ecological belonging in Indigenous and Western scientific treatments of self-transcendent emotions.

Indigenous sciences		Western science	
Kin relationality	Ecological belonging		
Provision of care to all forms of life	Provision of care to the larger environmental system	Provision of care to vulnerable offspring	Compassion
Reverence for all relations independent of collaboration	Responsibility toward the environmental system	Appreciation of gift from trading partner	Gratitude
Familial association to all forms of life and phenomena	Sense of belonging to the larger planetary system	Feeling connected to things larger than the self	Awe

Recent work has revealed a common structure to the experience of awe across over 25 cultures (Keltner et al., 2022). Across several cultures awe is communicated in similar fashion in specific vocalizations (Cordaro et al., 2016) and in patterns of head, gaze, and facial activity when encountering stimuli such as fireworks (Cowen et al., 2021). It is noteworthy that awe is evoked in encounters with many cultural forms, including ritual, music, visual design, and certain religious or spiritual practices (Keltner, 2023), which themselves bring about strong integration of the individual into collectives (Savage et al., 2021). In the next section we elaborate upon this, arguing that within both Indigenous and Western scientific perspectives, cultural practices cultivate the self-transcendent emotions to bring about a deeper sense of kin relationality and ecological belonging.

The neuroscientific study of awe has focused on the default mode network, or DMN, cortical regions engaged when people process information from an egocentric point of view (Hamilton et al., 2015). In a study from Japan, one group of participants watched videos of awe-inducing nature (footage of mountains, ravines, skies, and animals from BBC’s Planet Earth). Other participants viewed more threat-filled awe videos of tornadoes, volcanoes, lightning, and violent storms (Takano and Nomura, 2022). Both led to reduced activation in the DMN (see also Elk et al., 2019). The positive form of awe led to increased connections between the DMN and a region of the brain (the cingulate cortex) involved in our sense of reward. Threat-based awe led to increased connections between the DMN and the amygdala, which activates fight-or-flight physiology.

We also note that self-reports of awe and gratitude also covary with reduced levels of Interleuken 6, a biomarker that tracks activation of the cytokine system (Stellar et al., 2015). This finding is intriguing given associations between elevated inflammation and loneliness, social rejection, and shame, all mental states associated with separating from collectives (Hawkley and Cacioppo, 2010; Moieni and Eisenberger, 2020). Feelings of gratitude and awe, by contrast, are associated with the sense of social integration, or kin relationality, which reduces inflammation. Awe is more reliably associated with a particular kind of “chills,” which reflects the piloerection of muscles surrounding hair follicles in the arms and neck, and is thought to be associated with the individual merging with the collective (Campos et al., 2013).

Both Indigenous and Western perspectives highlight the central role of transcendent states in guiding prosocial behavior. We would also anticipate many cross-cultural similarities in these self-transcendent emotions across Indigenous and non-Indigenous cultures globally. At the same time, the Indigenous concepts of kin relationality and ecological belonging reveal straightforward ways in which these two central ethical concepts give rise to a cultural shaping of these emotions, very much in keeping with cultural approaches to the emotions (e.g., Mesquita and Frijda, 1992). We highlight several possibilities in Table 2.

Most notably, as one can see in Table 2, within Indigenous conceptualizations of kin relationality, experiences of compassion, gratitude, and awe are extended to all living forms within interdependent relations. We would further expect experiences of self-transcendent emotions in Indigenous cultures, more so than Western ones, to be more regularly oriented toward Earth systems, for example, local ecosystems, natural elements, and flora and fauna.

Extending these speculations, consider likely cultural variations in compassion. Within Indigenous cultures, kin relationality entails that self-transcendent states like compassion are extended to the broadest scope of entities, from genetic relatives, narrowly defined, to fellow group members, to the whole cosmos. By contrast, Western science has long emphasized genetic relatedness as the basis of compassion. Caring for offspring is a community responsibility in Indigenous societies —not only a parental one, like in many Western cultures. In view of this, it is not surprising that compassion responses are more commonly observed in Indigenous communities in the face of deepening inequalities, to strengthen community-based responses to care (Stellar et al., 2012; Rivera and International Labour Organization, 2020).

The framework of ecological belonging further reveals likely cultural influences upon compassion within many Indigenous cultures: This emotion would orient toward caring for social and ecological systems within Indigenous cultures. Such compassion for non-human entities is largely ignored in Western approaches to the emotion (e.g., Goetz et al., 2010). From an Indigenous perspective, flourishing depends on environmental well-being. There is no human flourishing without planetary health. This extension of compassion-related caring to all life forms, central to the two Indigenous concepts reviewed, is an uncharted and fascinating area of research that would be well served by the methods of Indigenous and Western sciences we have outlined above.

## The cultivation of self-transcendence: indigenous and western scientific perspectives

Central to Indigenous scientific perspectives is the idea that phenomena are cultivated within the fabric of culture—like kin relationality and ecological belonging, or self-transcendent states like compassion, gratitude, and awe. Ethical principles and the emotions that sustain them are observed within relational and communal social interactions and contained in oral, embodied, and collective narratives, as we suggested above. Subjective life is shared, contextual, and embodied.

Recent approaches to cultural evolution converge with this thinking. It is assumed that culture can be thought of as an ever-evolving repository of shared knowledge, experience, and practice, enabling social tendencies beneficial to the group (Boyd and Richerson, 1995; Henrich, 2016; Henrich et al., 2016). Very much convergent with assumptions of Indigenous sciences, it is reasoned that cultural practices like stories, rituals, ceremonies, jurisprudence, and lifeways enable the cultivation, experience and expression of self-transcendent emotions for the benefit of the collective (Keltner et al., 2022; Keltner and Oatley, 2022). These converging insights offer a framework for studying the cultural processes that transform ethical principles like kin relationality and ecological belonging, and transcendent states like compassion, gratitude, and awe, in ever-changing processes of cultural evolution.

Kin relationality and ecological belonging are among the highest qualities for community-building and leadership in Indigenous societies. These self-transcendent frameworks are transmitted and strengthened through several cultural processes, including intergenerationally. Within these practices of relating and interconnecting are woven ideas of belonging to group identity in developing life stories (Celidwen, 2021).

Thus, narratives are vessels for integrating prosociality into the culture through practices that extend care and ideas of personhood to landscapes and bodies of water, much as those that protect vulnerable populations (Lokensgard, 2018; Dudgeon and Bray, 2019; Burns et al., 2021; Celidwen, 2022a). For example, the Arctic Dene Peoples of Dakelh practice continuous reciprocity consisting of detailed and complex expressions of gratitude to sacred landscapes in recognition of the interdependence with the environment (Redvers et al., 2020).

Indigenous scholars emphasize the perception of self and world as preserved and transmitted through narratives (Cajete, 2005; Kovach, 2009; Smith, 2012). Stories serve as pedagogical means to cope with challenges (Marshall, 2002). They have been adopted as an alternative methodology in recent initiatives for social and environmental justice (Caxaj, 2015; Datta, 2018; Kraft and Johnson, 2018) and are at the core of the repositioning of Native pedagogies, science, philosophy, and spirituality (Hodge et al., 2002).

Cosmogonies, so central to culture, engender belonging and responsibility to the larger system, evocative of feelings of compassionfor the Earth community, gratitude, wonder, awe, and reverence for life. As the seeds from which the universe and cultures originate (*kosmos*: order, *gonos*: seed), these stories establish lifestyles and ways of assessment and experience. Accordingly, the ritual performance— so rich in eliciting awe—is an embodiment of narratives to heighten the incorporation of cultural values in relationships.

Origin stories help to achieve an understanding of the collective environment, along with the social conventions construed around it (Cajete, 2009; Colquhoun and Dockery, 2012; Datta, 2018; Charles and Cajete, 2020). These narratives are portals to subjective and collective experiences. Thus, everyday interactions express the shared experiences with embedded principles providing insight, inspiration, and motivation. As Indigenous identities are strongly connected to their natural territories, stories integrate changing aspects of everyday environmental occurrences and cycles (Celidwen, 2021).

When foundational stories are lost through colonization or globalization, the channels of communication with group identity and individual purpose are also lost; given our arguments here, the loss of such cultural activity could undermine the cultivation of self-transcendent states and prosociality (Celidwen, 2017). This undermining of transcendent states and prosociality could readily give rise to habitat degradation and extractive and exploitative practices. A limited sense of belonging instigates alienation, antisocial behavior of hostility and dominance, and helplessness and isolation. This loss may result in further confrontation and oppression of marginalized groups.

Indigenous Peoples recover and recontextualize stories in ongoing co-creation and participation, thus strengthening identity and purpose, and restoring community bonds. These stories, still oriented toward reverence to all living forms, encourage empathy and perspective taking, bringing individuals into resilient and adaptive communities. These dynamics of Indigenous stories are consistent with research showing that storytelling and journaling lead to well-being and health benefits, such as stress reduction and reduced depression, especially when recovering from trauma (Pennebaker, 2004).

Stories have been the preferred means to communicate shared identities of transcendence in kin relationality and ecological belonging. Life stories develop from cultural understandings that foster transcendent identities, where compassion extends to all phenomena, gratitude is expressed without expectation, and awe grows into reverence toward all of life. Imbued with these two core concepts, Indigenous narratives bring transcendent meaning to experiences of compassion, gratitude, and awe.

A raising of the profile of the robust Indigenous concepts of kin relationality and ecological belonging points to paths by which compassion, gratitude, and awe may be refined to meet the crises of our times.

## Implications of kin relationality and ecological belonging for psychological science

Psychological science has advanced profoundly by looking to the psychologies of people from cultures other than WEIRD groups. Doing so has led to an understanding of how culturally rich concepts, like the interdependent self or culture of honor influence thought, feeling, and action. Indigenous cultures have only recently begun to be systematically considered within psychological science. Given this state of affairs, we have outlined the centrality of two constructs in Indigenous science—kin relationality and ecological belonging. We have detailed how these core concepts transform self-transcendent emotions in striking ways. These concepts and their connections to emotions like compassion, gratitude, and awe are cultivated in culturally rich stories, rituals, and ceremonies, giving rise to cultural variations in prosociality.

The study of kin relationality and ecological belonging from Indigenous and Western scientific perspectives represents an exciting new frontier in cultural psychology with implications for the study of other phenomena than prosociality and self-transcendent emotions. For example, considerable progress has been made in understanding the interdependent (or collective self), that across cultures the self is construed as linked to other people (and not separate), similar to others (and not distinct), and can lead to healthier outcomes, to sharing, and that subjective experiences and ensuing actions are influenced by other people (Markus and Kitayama, 1991; Walker et al., 2005; Cross et al., 2011; Vignoles et al., 2016; Chin, 2019; Salvador et al., 2020; Bhattarai et al., 2022). A critical question for this literature is Indigenous kin relationality. We suggest, building on the insights of others, that an Indigenous self-construal does not simply reduce to an interdependent self. For example, within the broader value systems of kin relationality and ecological belonging, the self is more linked to Nature and her systems and cycles; it shares fundamental properties of the natural world; and the individual’s sense of how actions and emotions arise are shaped by the influence of natural forces. Given the arguments we have offered here, we would expect the Indigenous self to be shaped more directly by environmental processes, such as the health of bodies of water, forests, and soils, and more directly influenced by community participation. Little if any attention has been given to these possibilities in the literature on the interdependent self.

As living relations are built through intergenerational adaptation among peoples, human, other-than-human, and environmental, which form the dimensions of identity, the concept of self is necessarily made of relationships that build biological, cultural, and political identities. Thus, Indigenous kin relationality, we further note, is highly contextual, and is found in meaning-making in relation to Lands and their specific phenomena. Still, within this complex system, at a global level, Indigenous perspectives share remarkably similar views in conceiving the planet as a Mother and all creation as related, and share a sense of kin and belonging within a caring and responsive environment. More generally, we note that within the Indigenous kin relational self, people do not view themselves as being *in* a relationship with other people in human-only social contexts or things; instead, the individuals perceive themselves as *being* the relationships that are held and formed, and actively *becoming* the integrated systems of belonging with human beings, other-than-human beings, lands and ecosystems, and cosmic systems and phenomena.

The study of well-being represents another important area whose study kin relationality and ecological belonging will inform. Simply put, within Indigenous frameworks, and very much in keeping with kin relationality and ecological belonging, human well-being is intertwined with the well-being of ecosystems; there is no human flourishing in Indigenous cultures that is separate from planetary flourishing. Western psychological science has indeed documented that human well-being benefits when individuals relate more extensively with nature (Kuo, 2015; Hansen et al., 2017; Yu et al., 2017; Grabowska-Chenczke et al., 2022), and, of course, human actions are harming the natural world (and protecting it). Our analysis of kin relationality and ecological belonging highlights several intriguing possibilities. We would expect people within Indigenous cultures to conceptualize their well-being more in terms of the health of ecosystems, to fluctuate more in their well-being in terms of changes to the natural world, and to be more acutely aware of how their actions influence planetary health. We note that no approach to well-being in Western Science measures or models comprehensively this intertwinement of humans and the natural world in terms of stress, flourishing, thriving, or happiness. We suggest this is an important area of inquiry, one that flows directly out of Indigenous concepts of the Ethics of Belonging.

## Author contributions

YC worked on the conceptual analysis, original draft, and led the research and writing of Indigenous approaches to self-transcendence. DK led the research and writing of the neurophysiology of compassion, gratitude, and awe. YC and DK worked on revisions. All authors contributed to the article and approved the submitted version.

## Footnotes

^1^In this essay, we build upon the concept of *homo reciprocans* referred to the tendencies to cooperate and reciprocate (Dohmen et al., 2009) by detailing the psychological processes that enable people to transcend the transactional relationship between individuals and the expectation of receiving through the lens of the Indigenous “ethics of belonging” and its concepts of kin relationality and ecological belonging.
